# Protein dysregulation in graft versus host disease

**DOI:** 10.18632/oncotarget.23276

**Published:** 2017-12-15

**Authors:** Liren Qian, Delia Dima, Cristian Berce, Yu Liu, Ioana Rus, Lajos-Zsolt Raduly, Yi Liu, Bobe Petrushev, Ioana Berindan-Neagoe, Alexandru Irimie, Alina Tanase, Ancuta Jurj, Jianliang Shen, Ciprian Tomuleasa

**Affiliations:** ^1^ Department of Hematology, Navy General Hospital, Beijing, PR China; ^2^ Department of Hematology, Ion Chiricuta Oncology Institute, Cluj Napoca, Romania; ^3^ Iuliu Hatieganu University of Medicine and Pharmacy, Cluj Napoca, Romania; ^4^ Department of Stem Cell Transplantation, Fundeni Clinical Institute, Bucharest, Romania

**Keywords:** allogeneic stem cell transplantation, biomarkers, proteins, graft versus host disease, exosomes

## Abstract

Allogeneic hematopoietic stem cell transplantation is a well-established treatment for many malignant and non-malignant hematological disorders. As a frequent complication in up to 50% of all patients, graft-versus-host disease is still the main cause for morbidity and non-relapse mortality. Diagnosis is usually done clinically, even though confirmation by pathology is often used to support the clinical findings. Effective treatment requires intensified immunosuppression as early as possible. Although several promising biomarkers have been proposed for an early diagnosis, no internationally-recognized consensus has yet been established. Protein-based biomarkers represent an interesting tool since they have been recently reported to be an important regulator of various cells, including immune cells such as T cells. Therefore, we assume that protein dysregulation is important in the pathogenesis of acute graft versus host disease and their detection might be an possibility in the early diagnosis and monitoring. In this review, we aim to summarize the previous reports of protein biomarkers, focusing on the pathogenesis of the disease and possible implications in diagnostic approaches.

## INDICATIONS FOR STEM CELL TRANSPLANTATION

**Figure 1:**
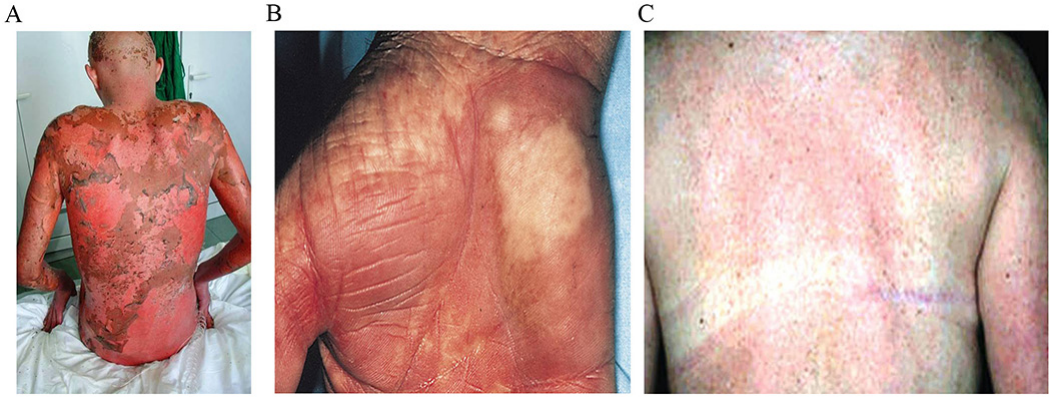
**(A)** Acute stage IV GVHD, following a myeloablative conditioning chemotherapy. **(B)** Chronic skin GVHD, following conditioning chemotherapy with TBI and cyclophosphamide. **(C)** Chronic GVHD, following reduced intensity conditioning chemotherapy.

Hematopoietic stem cell transplantation (HCT) is an efficient immunotherapy for blood malignancies. HCT relies on exploiting the graft versus leukemia (GVL) effects of allogeneic cells [1–3]. Allogeneic HCT (allo-HCT) is commonly used for acute myeloid leukemia (AML), with 30% of allo-HCTs are used in patients with AML, 15% in patients with acute lymphoblastic leukemia (ALL), 15% in patients with myelodysplastic syndromes and 10% in patients with non-Hodgkin’s lymphomas [4, 5]. The rest 30% of allo-HCTs are carried out for patients with chronic lymphocytic leukemias (CLL), plasma cell disorders, hemoglobinopathies, thalassemia, primary immunodeficiency and autoimmune diseases [6, 7]. Disease stage at the time transplantation is performed is of key importance because of relapse risks, as well as toxicity concerns and its main complication: acute graft-versus-host disease (GVHD) (Figure 1). For acute leukemias, HCT is carried out after the first or second complete remission and for lymphoid malignancies, allogeneic SCT is a viable option after the failure of several chemotherapy lines. In the case of myelodysplastic syndromes, therapeutic guidelines are in favor of waiting for disease progression until the phase of blast excess before transplantation is done in order to achieve a higher survival rate [8–10].

For a HCT, the selection of the donor is maybe the most important aspect of the pre-transplantation procedures and human leukocyte antigen (HLA) compatibility plays a greater role than even in solid organ transplants. All the genes that encode the HLA antigen are situated on chromosome 6 and thus, the probability of a sibling with a HLA identical match is 25%. Still, without a compatible relative, patients make use of unrelated donors, with the International Donor Registry now reaching around 20 million HLA-types donors [11, 12]. Caucasian patients have the best chances of finding a HLA-suitable donor for class I and II loci, estimated at about 60% [13, 14]. A half-matched donor is certainly an option, but due to the haploidentical transplant, an intensive cell deletion is required. The results are worse than in a matched transplant with a reduced graft-versus-tumor outcome that results in graft rejection, increased infection risks and malignancy relapse [15–17].

Apart from the HLA-typing, the pre-transplant procedures include chemotherapy and total-body irradiation in order to achieve myeloablation and later allow the donor stem cells to restore the patient’s immune system and shorten aplasia. Current protocols use reduced-intensity regimens (RIC) that remove myeloablative chemotherapy and still allow the intensity to be high enough to avoid graft rejection. RIC has also improved stem cell transplantation limitations, making this technique available for the ones over 60 or even 70 years, the group that has the higher prevalence of hematological malignancies [18–20].

## EXOSOMES AS THE BACKBONE OF INTERCELLULAR COMMUNICATION

In a malignant tumor, cells can grow without needing the involvement of the surrounding microenvironment for a limited period of time [21–23]. Afterwards, the malignant cells act at the tumor-vascular interface where they orchestrate the recruitment of endothelial, perivascular or inflammatory cells, as well as platelets and clotting factors [24]. This will alter the local vascular homeostasis. The interaction is mediated by various cytokines, growth factors, proteins or even more important, microRNA species. MicroRNAs (miRs) are short, non-coding RNA species that have been proven to activate messenger RNA (mRNA) in normal development and disease [25–28]. MiRs are either released by cells protected from the surrounding environment in a variety of vesicles or are associated in complexes with proteins. The package through which cells communicate with the outside world by using miRs is extremely important and exosomes were the first extracellular vesicles shown to contain microRNAs [29–32].

Membrane release into the extracellular space has been known and described by Black et al some years ago [33], but only recently it has been proven that microvesicles released by cells are indicative to the cell type or its function. These micro-structures are important in cell-cell transfer and communication and are important in physiological and pathological processes, being found in all body fluids and binding only to selected targets [34]. There are various types of such vesicles, based on their different origin, biogenesis and function. Endosomes and lysosomes undergo a physiological process similar to exocytosis and fuse their membranes with the plasma membrane [35, 36]. This will lead to the expression of the surface of the vesicle of certain receptors that are also found in the endoplasmic reticulum [37].

Based on their size, the major vesicle populations may be exosomes, microvesicles or apoptotic bodies [38]. Extracellular vesicles are depicted as unique “messenger” used in cell-to-cell communication and mediate the trafficking of various molecules that are traditionally regarded as either insoluble or cell-associated. Such molecules include various membranes, cytoplasmic or nuclear proteins, as well as nucleic acids [39]. Various researchers worldwide have isolated RNA from circulating cancer cells [40] or from bodily fluids [41]. If we consider that RNA is easily degraded and has a short half-life if unprotected in serum [42], it is very fair of us to deduce that the majority of the cell-free RNA species is harbored in different exosomes or similar microvesicle fractions. MicroRNAs may also be encapsulated by protein complexes in blood [43, 44].

As such vesicles are carriers for many molecules between human tissues, they may be considered as potential new sources of biomarkers. Consistent to their physical and immunological characteristics, that include the presence of proteins like Rab GTPases, annexins, flotillin, Alix or Tsg101, as well as integrins or tetraspanins (CD63, CD9, CD81 or CD82), apart from lipids like cholesterol, ceramide or sphingolipids [45, 46], we can describe their synthesis. They are generated by reverse budding of the membrane of multivesicular bodies (MVB) [39, 47, 48], that fuse with the plasma membrane previously liberating these particles outside the cell. Then, exosomes enter the vascular or lymphatic system and circulate freely until they bind to their specific target. The release from the cell is possible after the membrane is stimulated by calcium ionophores or phorbol esters. After the synthesis, that can be done in as little time as three hours [49], such structures are released and may either taken by the systemic blood stream, or may remain in the extracellular space near the cell of origin. After being released, they diffuse into bodily fluids such as blood, urine, amniotic fluid, saliva, lung surfactant, malignant effusions, breast milk or even semen. A common exosomes may include hundreds of proteins [30] and influence the fate of other tissues. Examples include annexins and alter the dynamics of the cytoskeleton, Rab GTPases and promote docking and membrane fusion events or endosomal sorting complexes desired for transport [50–52]. In hematopoietic tissues, exosomes express on their surface high MHC class I or II molecules [53–55] and various adhesion molecules like CD146, CD9, EGFRvIII, CD18, CD11a, CD11b, CD11c, CD166 or LFA-3/CD58 [56, 57]. Specific surface markers allow exosomes to be absorbed by distant cells and transfer their proteins, mRNAs, microRNAs, lipids or any other types of molecules, thus forming a particle-based endocrine system.

## PROTEIN DYSREGULATION IN HEMATOLOGICAL MALIGNANCIES AND GVHD

The studies included in the present paper were identified after a search of the National Library of Medicine’s MEDLINE database using PubMed and Google Scholar. Candidate papers were limited to English, German, and French language publications, but were not limited to any geographical region, and the most recent search was performed on August 28th, 2017. Only papers published between 1990 and 2017 were considered in order to avoid any inconsistencies in diagnostic criteria and also cover the period of publications on opioid rotation. The search strategy was based on the combination of the keywords “allogeneic stem cell transplantation”, “biomarkers”, “hematology”, “proteomics”, and “protein dysregulation”. Subsequently, an additional manual search of the citations of the previously selected papers was performed.

GVHD progression is characterized by the differentiation of various alloreactive T cells into effector cells as a result of the synthesis of additional inflammatory cells and a high cytokine dysregulation [58, 59]. The same dysregulation caused by GVHD leads to a suppression of the immune system and to tissue damage in various organs such as liver, skin or intestinal mucosa. IL-6 is one of the most important protein-based molecules as it has an inhibitory role over the recognition of the regulatory T-cells after hematopoietic stem cell transplantation. Chen et al [60] have proven this hypothesis by showing that an antibody-linked blockade of the IL-6 receptor serves to recalibrate the effector and regulatory arms of the immune system and represents a novel, potentially clinically translatable, strategy for the attenuation of GVHD. Min et al [61] further expand this study and conclude that a high serum IL-6 level one week post-transplantation may be an early predictor of transplant-related complications and that it seems to trigger pro- and anti-inflammatory cytokine release. Kinetic patterns of IL-6 and IL-10 were more exaggerated in those with complications after HSCT. The Th2 cytokine IL-10 plays a major inhibitory effect on Th1 cells and their potential in cytokine production, but various studies show that a direct correlation exists between a high level of IL-10 and transplant-related complications. This is linked to balancing the immune system in HSCT. The direct correlation further links a high IL-10 concentration in the serum of transplant patients to the levels of various inflammatory cytokines such as IL-6, IL-1, that can thus be associated with a high grade of hepatic injury, as shown by Yeh et al [62]. Recent data states that IL-15 is another molecule whose expression or dysregulation is linked to the administration of exogenous interleukin-15, of great importance in ontogeny, as well as in the underlying mechanisms of GVHD as a result of their interference with allogeneic T cells. Choi et al [63] stated that an increased level of TNF-alpha is associated with the development of GVHD as high levels of TNF alpha induce the activation of apoptotic cells and produce an allo-antigenic reaction, that is directly linked to the appearance of severe tissue damage via apoptosis and necrosis. The group of Yamamoto et al [64] also correlate the early expression of plasmatic CC-chemokine ligand motif 8 (CCL8) to GVHD and taken together with an involvement of allo-recognition in CCL8 expression, it suggests that CCL8 plays an important role in GVHD pathology, but more detailed analysis and further experiments are required to draw a clear conclusion.

Proteins can be used as biomarkers for various diseases, including in hematology and oncology. As GVHD is an immune-mediated disease, that can target organs in the human body, it represents on the most serious clinical problems in allogeneic stem cell transplantation due to its high rate of mortality and morbidity [65, 66]. For the past few years, physician scientists have tried to develop new therapeutics in order to customize the delivery of new immunosuppressive agents as to obtain the optimal results regarding patient care. Such research was focused on the discovery and validation of GVHD-related essential biomarkers with the main purpose to predict and evaluate the response to therapy, as well as to understand the complex pathophysiology of GVHD. Proteomics is a field with a huge potential impact in the study of GVHD and previously-published papers confirm this hypothesis [58, 67, 68].

The use of various biomarkers for assessing GVHD, may was investigated by various physicians lately, including Magenau et al [69, 70]. The optimization of the prevention and therapy of GVHD is most likely to improve the therapeutical outcome of allogeneic stem cell transplantation, with the gradual introduction of a targeted approach in GVHD prophylaxis. This approach is based on translational research, with a special emphasis on the central interaction between antigen presenting cells and T lymphocytes, with regulatory T cells maintaining a peripheral tolerance and targeting main transcriptional and signaling pathways of T cells.

Liao et al [71] have proven that IL-2, a molecule that is of key importance in GVHD, influences helper T-cell differentiation by modulating the expression of cytokine receptors in order to help specify and maintain the differentiation status of the immune cells. Cheraï et al [72] have confirmed the results of Liao et al by proving that natural regulatory T-cells are of great therapeutic potential in inducing tolerance in allogeneic cells and organ transplantation. This is achieved as IL-2 influences helper T-cell differentiation by modulating the expression of various cytokine receptors and maintaining the differentiation status of the immune cells. Intrleukin-6 (IL-6) is another molecule of key importance in GVHD as it is correlated with inflammation and maturation of B-cells and acts as a pro-inflammatory cytokine or as an anti-inflammatory cytokine. Massaro et al [73] proved correlated post-transplant death with IL-6, procalcitonin and C reactive protein (CRP). These results were later confirmed by Pai et al [74] who proved reduced skin GVHD by bortezomib was correlated with reduced serum and skin IL-6 levels. Thus, the administration of a blocking IL-6 antibody in this model also resulted in similar cutaneous GVHD protection. These results indicate that bortezomib or blockade of IL-6 may prevent CD8(+) T cell-mediated cutaneous acute GVHD. Apart from IL-6, IL-8 seems to be important as a biomarker for GVHD, as proven by Pidala et al [75], as well as by Bergen et al [76]. The Italian group has proven that even if the serum cytokine levels were related to several variables associated with HSCT, the two cytokines IL-8 and IL-2Rα are predictors of GVHD II-IV and III-IV, translating into a higher treatment-related mortality (TRM) risk (17% vs 3%, P=0.004). IL-10 is another protein involved in GVHD pathogenesis, as it is another anti-inflammatory cytokine that down-regulates the expression of the Th1 cytokine, as well as of the major histocompatibility (MHC) class II antigens and their co-stimulatory molecules in macrophages, just like IL-12hi [77, 78].

## ROLE OF EXOSOMES IN PROTEIN DYSREGULATION AND GVHD

One third to almost half of the patients that undergo an allogeneic stem cell transplantation develop either acute or chronic GVHD, out of which most of them are difficult to treat using corticosteroids [79]. Third party mesenchymal stem cells (MSC) have been presented as one possible treatment option for these cases [80], being immunosuppressive and having the ability to prolong the mismatched skin grafts in various mammalian models. Still, new data rather suggest that MSCs have a beneficial effect over the therapy-refractory MSC not because of their direct action, but due to the secretion of various modulating factors [81, 82]. These soluble molecules, of great clinical applications, are secreted *ex vivo* and are transported in the bodily fluids through small, extracellular vesicles that bud from the plasma membrane. Such vesicles are known as exosomes [83]. The exosomes are released by exocytosis upon fusion of the multivesicular bodies with the plasma membrane and contain lipids, RNA, small non-coding RNAs, as well as proteins [84–90]. All of these factors are transported through the blood stream within exosomes and certainly contribute to inter-cellular communication, thus exerting various immune stimulatory or immune suppressive functions. In a recent paper of the German Cancer Consortium (DKTK - Deutsched Konsortium Translationale Krebsforsching) [91], they used exosomes secreted by mesenchymal stem cells to modulate the immune system and treat chronic GVHD. MSM from four different patients from four different cancer centers of the DKTK, that have underwent a matched unrelated donor (MUD) transplant, the conditioning media was harvested every 48 hours and afterwards the exosomes were isolated. These exosomes were analyzed in order to see whether they have any pro or anti-inflammatory activity, being used as apoptosis-inducing molecules. The exosomes were proven to transport interferon (IFN)-γ, interleukin (IL)-8, as well as IL-10, transforming growth factor (TGF)-1. Before being tested in patients, the immune modulatory potential was assessed on peripheral blood mononuclear cells (PBMC) and natural killer cells, in mixed lymphocyte reactions. In order to reproduce different allogeneic transplantation settings, HLA class I negative K562 cells, as well as stable HLA-E^*^01:03 or HLA-B27-transfected K562 variants were used as allogeneic target cells [92]. The MSC-derived exosomes stimulated the PBMC and NKs derived from the patients to secrete IL-1, tumor necrosis factor (TNF)-α and IFN-γ, proving the immunosuppressive effects of the exosomes and of their protein-based content. The next step was to treat chronic GVHD *in vivo* on a human subject. 0.4-9 x 10^6 MSCs are usually used to treat refractory chronic GVHD [93]. Exosomes obtained from 4 x 10^7 MSCs were isolated and calculated as the corresponding dosage for the body weight for the patient and defined as one unit. After four unites were administered, the clinical symptoms of chronic GVHD disappeared and no side effects were detected. Thus, the proteins transported through the blood flow within exosomes have been proven to be efficient in treating GVHD.

Raposo *et al* have proven that B lymphocytes release major histocompatibility complex (MHC) class II containing exosomes that induce MHC class II-restricted T lymphocyte responses [94], whereas Zitvogel have shown that dendritic cells release exosomes that carry various functional peptide-bearing MHC class I and class II molecules [95]. These vesicles promote immunosuppressive responses in mice and have the ability to directly induce the immune responses of CD8+ T lymphocytes, as well as to activate naïve CD4+ T lymphocytes in an antigen-specific manner. Exosomes often containing molecules that are associated with various immune suppressive functions, such as Fas ligand, can induce the apoptosis of an activated T cell [96]. Exosomes also mediate the down-regulation of NKG2D expression on NK cells, as well as CD8+ lymphocytes, which correlates with the functional impairment of both types of lymphocytes [97], with direct implications in transplant immunology, as proven by Pêche et al [98]. They aimed to analyze the effect of the presentation of donor MHC antigens from immature DC-derived exosomes post-transplantation using congenic rats fully mismatched for the class I and II genes of the MHC region. Exosomes were able to significantly prolong graft survival. But the effect was rather moderate. In order to ensure the presentation of allo-antigens, they have used the LF 15-0195 drug, a new desoxypergualin analog that has recently been reported to prevent DC maturation *in vivo* [99, 100]. The combination of donor-derived exosomes with a short-term treatment with the LF 15-0195 had synergistic effects and induced donor-specific allograft tolerance accompanied by prevention or a considerable delay of the appearance of chronic rejection.

If we consider exosomes as the therapeutically active component of a mesenchymal stem cell infusion, the number of clinical applications is comparable to the applications of MSC in transplant medicine. Even more, exosomes are non-self-replicating and because of their small dimensions, may be easily sterilized through filtration. Thus, their clinical potential, both as therapeutics and as diagnostics, is increased.

## FUTURE DIRECTIONS

In many cases, cancer diagnosis is too late or a very efficient treatment because there are no specific biomarkers that might allow doctors to identify a patient with early disease. This is also the case for detecting very early GVHD or disease recurrence or to monitor the tumor’s response to chemotherapy. Early detection of all these clinical settings is important to obtain excellent survival rates and current standard-of-care identifies the presence of malignancy after biopsy procurement. This technology is nevertheless sometimes very inaccurate and with important side-effects, highlighting the tremendous importance of minimally invasive management.

Exosomes from different tissues express specific markers on their surface, as presented earlier. These characteristics make them ideal future candidates for biomarkers and a highly efficient management of all diseases, from cancer to chronic inflammatory diseases or infections.
